# Optomechanically induced gain using a trapped interacting Bose-Einstein condensate

**DOI:** 10.1038/s41598-023-30573-4

**Published:** 2023-03-04

**Authors:** H. Mikaeili, A. Dalafi, M. Ghanaatshoar, B. Askari

**Affiliations:** 1 Laser and Plasma Research Institute, Shahid Beheshti University, Tehran, Iran; 2 Department of Physics, Shahid Beheshti University, Tehran, Iran

**Keywords:** Matter waves and particle beams, Quantum mechanics

## Abstract

We investigate the realization of the phenomenon of optomechanically induced gain in a hybrid optomechanical system consisting of an interacting Bose-Einstein condensate trapped inside the optical lattice of a cavity which is generated by an external coupling laser tuned to the red sideband of the cavity. It is shown that the system behaves as an optical transistor while the cavity is exposed to a weak input optical signal which can be amplified considerably in the cavity output if the system is in the unresolved sideband regime. Interestingly, the system has the capability to switch from the resolved to unresolved sideband regime by controlling the *s*-wave scattering frequency of atomic collisions. We show that the system gain can be enhanced considerably by controlling the *s*-wave scattering frequency as well as the coupling laser intensity while the system remains in the stable regime. Based on our obtained results, the input signal can be amplified more than 100 million percent in the system output which is much larger than those already reported in the previously proposed similar schemes.

## Introduction

In recent decades, ultracold atomic ensembles trapped in optical lattices generated by quantized light fields^1^ and hybrid optomechanical systems containing Bose-Einstein condensates (BECs)^2–5^, where the excitation of a collective mode of the trapped atoms plays the role of the vibrational mode of the moving mirror in a bare optomechanical system (OMS)^6^, have attracted much attention. Such systems have been known as a good platform for studying the interaction of light with matter in the regime where their quantum mechanical properties are manifested in the same level^7–11^.

The optomechanical coupling generated by an external coupling laser between the optical mode of the cavity and the fluctuation of the collective excitation of the BEC (the so-called Bogoliubov mode)^12,13^, makes the system behave effectively as a two-mode quantum system in which the phenomenon of optomechanically induced transparency (OMIT)^14–17^ can be observable while the cavity is also driven by another external weak probe laser. One of the most important features of a hybrid OMS containing a BEC is the nonlinear effect of atomic collisions which behaves as an atomic parametric oscillator^18–21^ which brings more controllability^22,23^ and can increase the quantum effects at the macroscopic level^24–26^.

A remarkable feature of the OMIT phenomenon is the possibility of slow and fast light realization^27–35^ which has important applications in quantum information and quantum communications. As is well-known in the standard OMIT phenomenon, the transmitted light intensity at the probe frequency in the cavity output is lower than or equal to that in the input where the equality (corresponding to $100\%$ probe transmission) occurs when the damping rate of the mechanical oscillator is much lower than the optical damping rate. Nevertheless, there is a very interesting case in which the transmitted probe amplitude in the cavity output is stronger than that in the input of the cavity. In such a situation, the system exhibits a special kind of OMIT known as optomechanically induced gain (OMIG) or optomechanically induced amplification, so that it can be used as an optical transistor^36–44^. More recently, it has been shown^45,46^ that the phenomenon of OMIG can occur in a bare OMS in the unresolved sideband (URSB) regime^47^ while the coupling laser frequency has been tuned on the red sideband of the cavity (the red detuned regime of optomechanics). Although OMIG can be also achieved in the blue detuned regime of optomechanics, but the important problem is that OMSs are rarely stable in the blue regime.

Motivated by the above-mentioned investigations, in the present work we study a hybrid OMS consisting of an interacting BEC whose cavity is pumped by a coupling laser responsible for generation of an optical lattice inside the cavity. The optical lattice provides an effective optomechanical coupling between the cavity optical mode and the Bogoliubov mode of the BEC through a radiation pressure interaction. It is also assumed that the cavity is exposed to an input optical signal which is modeled as a time-dependent perturbation (playing the role of a weak probe laser).

Our aim in the present work is to demonstrate that the present hybrid OMS can be used as an optical transistor which can amplify the input optical signal in the output of the cavity. It is explicitly shown that in the URSB and red detuned regimes of optomechanics, the present hybrid OMS exhibits much larger gain in comparison to the previously studied schemes^36–44^ while the system is stable. Furthermore, one of the most important advantages of the present scheme in comparison to the previous ones is the controllability of the system gain which can be manipulated not only through the coupling laser pumping rate but also through the *s*-wave scattering frequency of atomic collisions, which itself is experimentally controllable through the transverse trapping frequency of the BEC^48^.

The structure of the paper is as follows: In section “System Hamiltonian” the Hamiltonian of the hybrid OMS is introduced. In section “Dynamics of the system” the dynamical equations of the system are derived based on the Heisenberg-Langevin equations and the response of the system to the input signal, which behaves as a time-dependent probe perturbation, is obtained. Then, in section “Results and discussion” we present the behavior of the system response to the input signal and the cavity power reflection coefficient. Finally, the summary and conclusions are given in section “Summary and conclusions”.

## System Hamiltonian

**Figure 1:**
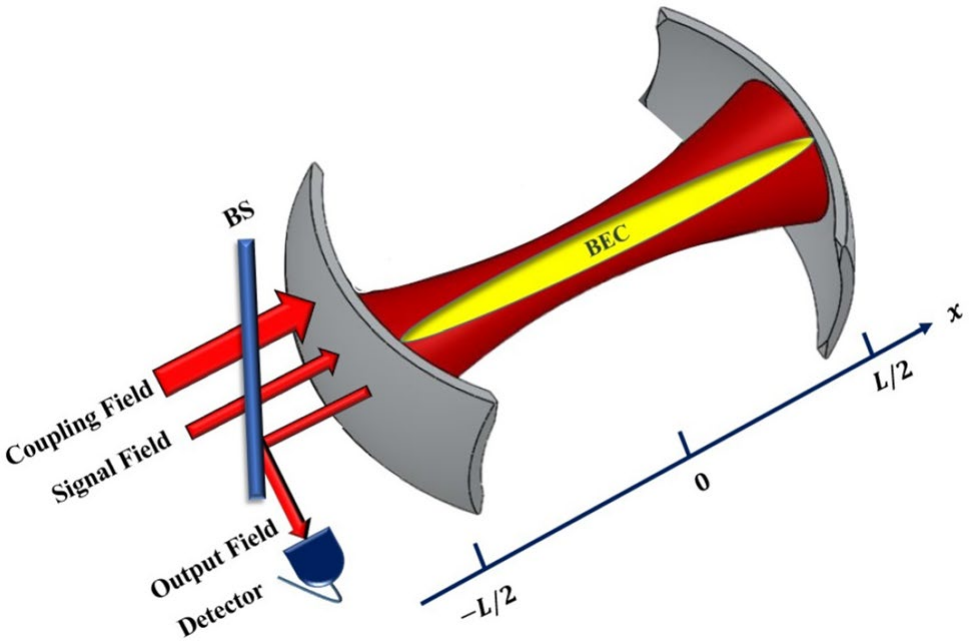
(Color online) A Fabry-Perot cavity containing a trapped BEC is driven by a strong coupling laser along its axis from the left mirror of the cavity where a weak input signal is also entered. The system behaves as an optical transistor that can amplify the input signal in the output which emanates from the left side of the cavity.

The studied system is a Fabry-Perot cavity with a length of *L* and a resonance frequency of $\omega _{0}$ containing a trapped one dimensional BEC as has been shown in Fig. 1. The BEC has been formed by *N* identical two-level atoms having transition frequency $\omega _{a}$ and mass $m_{a}$ confined in a cylindrically symmetric trap with a transverse trapping frequency $\omega _{\perp }$ and negligible longitudinal confinement along the x direction. The cavity is driven by a strong external (coupling) laser with a power of $P_c$ and frequency $\omega _{c}$ at the rate of $|\varepsilon _c|=\sqrt{2\kappa _e P_c/\hbar \omega _c}$ along its axis from the partially transparent mirror on the left side of the cavity which is responsible to produce a radiation pressure coupling with the matter field of the BEC. In order to see how the present system acts as an optical transistor, it is assumed that a weak optical signal (playing the role of a probe laser) with frequency $\omega _{s}$ enters the cavity as an input coherent field through the left mirror at the rate of $\varepsilon _s$.

In the dispersive regime, where the difference between the frequency of the coupling laser and that of the atomic transition is much larger than the atomic linewidth ($\Gamma _{a}$), i.e., $\Delta _{a}=\omega _{c}-\omega _{a}\gg \Gamma _{a}$, the excited electronic state of the atoms can be adiabatically eliminated and spontaneous emission can be ignored^49,50^, so that the second quantized Hamiltonian of the system can be written as

1 $$\begin{aligned} \hat{H}= & {} \hbar \omega _{0} \hat{a}^{\dagger }\hat{a}+\int _{-L/2}^{L/2} dx \hat{\Psi }^{\dagger }(x)\bigg (\frac{-\hbar ^2}{2m_a}\frac{d^2}{dx^2}+ \hbar U_0 \cos ^2(kx) \hat{a}^{\dagger }\hat{a}+\frac{1}{2} U_{s}\hat{\Psi }^{\dagger }(x)\hat{\Psi }(x)\bigg )\hat{\Psi }(x)\nonumber \\&+i\hbar \bigg (\varepsilon _c e^{-i\omega _c t}\hat{a}^{\dagger } - \varepsilon _c^{*} e^{i\omega _c t}\hat{a}\bigg )+i \hbar \bigg (\varepsilon _s e^{-i\omega _s t}\hat{a}^{\dagger } - \varepsilon _s^{*} e^{i\omega _s t}\hat{a}\bigg ), \end{aligned}$$

where ${\hat{\Psi }}(x)$ represents the atomic field annihilation operator in position space and ${\hat{a}}$ indicates the cavity mode annihilation operator in momentum space. Besides, $k=\omega _c/c$ is the wave number of the intracavity optical field, $U_0 =g_0^2/\Delta _a$ is the depth of the optical lattice potential per single-photon, $g_0$ is the vacuum Rabi frequency, $U_s =4\pi \hbar ^2 a_s/m_a$ is the strength of the atom-atom scattering wherein $a_s$ is the length of the two-body s-wave scattering.

If the condition of $U_0 \langle a^{\dagger }a\rangle \le 10\omega _R$ is satisfied, in which $\omega _R=\hbar k^2/2m_a$ is the atom recoil frequency, the system is in weak optical lattice regime. Under this condition and using the Bogoliubov approximation, the matter field ${\hat{\Psi }}(x)$ can be expanded as^51^

2 $$\begin{aligned} {\hat{\Psi }}(x) = \sqrt{\frac{N}{L}} + \sqrt{\frac{2}{L}}\cos (2kx) \hat{c}, \end{aligned}$$

where $\hat{c}$ is the BEC’s first excited mode annihilation operator in the momentum space satisfying the commutation relation $[\hat{c},\hat{c}^{\dagger }]=1$.

By substituting Eq. (2) into Eq. (1), the system’s Hamiltonian in the frame rotating at the coupling laser frequency can be rewritten as

3 $$\begin{aligned}&{\hat{H}} = \hbar \delta _{c} \hat{a}^{\dagger }\hat{a}+ \hbar \zeta \hat{a}^{\dagger }\hat{a} \hat{Q} + \frac{1}{2} \hbar \Omega _{c}\left( \hat{P}^2+\hat{Q}^2 \right) +\frac{1}{2} \hbar \omega _{sw} \hat{Q}^2+ i \hbar \left( \varepsilon _c \hat{a}^{\dagger } - \varepsilon _c^{*} \hat{a}\right) + i \hbar \left( \varepsilon _s e^{-i\delta t} \hat{a}^{\dagger } - \varepsilon _s^{*} e^{i\delta t} \hat{a}\right) , \end{aligned}$$

where $\delta =\omega _s-\omega _{c}$ is the detuning between the frequencies of the coupling and signal lasers. Besides, $\hat{Q}=(\hat{c}+\hat{c}^{\dagger })/\sqrt{2}$ and $\hat{P}=(\hat{c}-\hat{c}^{\dagger })/\sqrt{2}i$ are, respectively, the position and momentum quadratures of the BEC satisfying the commutation relation $[\hat{Q},\hat{P}]=i$. Furthermore, $\delta _{c}=\Delta _c + \frac{1}{2} N U_0$ is the Stark-shifed cavity frequency due to the presence of the BEC in which $\Delta _{c}= \omega _0 - \omega _{c}$ is the cavity resonance detuning with the frequency of the coupling laser. In fact, the presence of the BEC makes the bare resonance frequency of the cavity be changed from $\omega _{0}$ to $\omega _{0} + \frac{1}{2} N U_0$. As is seen from the second and third terms of Eq. (3), the BEC behaves as a mechanical oscillator (the so-called Bogoliubov mode) with the frequency $\Omega _{c}= 4 \omega _{R} + \omega _{sw}/2$ that has been coupled to the optical mode of the cavity through the optomechanical coupling parameter of $\zeta =\frac{1}{2}\sqrt{N}U_0$. Besides, the effect of atom-atom interaction with the *s*-wave scattering frequency $\omega _{sw}=8\pi \hbar a_{s}N/m_{a}Lw^2$ leads to the manifestation of the fourth term in Eq. (3), where *w* is the waist radius of the optical mode.

## Dynamics of the system

In this section, we study the dynamics of the system with the Hamiltonian of Eq. (3) in the framework of open quantum systems which is described by the following Heisenberg-Langevin equations of motion

4a $$\begin{aligned} \frac{d \hat{a}}{dt}= & {} -(\kappa + i\delta _{c}){\hat{a}} - \zeta \hat{Q}\hat{a} + \varepsilon _c + \varepsilon _s e^{-i\delta t} + \sqrt{2\kappa _e}{\hat{a}}_{in}+ \sqrt{2\kappa _i}{\hat{a}}_{int}, \end{aligned}$$

4b $$\begin{aligned} \frac{d\hat{P}}{dt}= & {} -\gamma _B \hat{P} -(\Omega _{c}+\omega _{sw})\hat{Q} -\zeta \hat{a}^{\dagger }\hat{a} + {\hat{P}}_{in}, \end{aligned}$$

4c $$\begin{aligned} \frac{d\hat{Q}}{dt}= & {} \Omega _c \hat{P}, \end{aligned}$$

where $\sqrt{2\kappa _e}{\hat{a}}_{in}$ and $\sqrt{2\kappa _i}{\hat{a}}_{int}$ are the input and internal noises, which are respectively originated by the input-output coupling and non-perfectness of the mirrors or light scattering of the remaining air molecules inside the cavity ($\kappa _i$ is the cavity internal decay rate). In principle, the dissipation of the intracavity field takes place in two different ways: the first one is the leakage that occurs through the left mirror with the rate of $\kappa _e$ which appears as the output field of the cavity that is detected on the left mirror, and the second one is the loss that occurs through inaccessible channels with the rate of $\kappa _i$. Therefore the cavity field amplitude damping rate is $\kappa =\kappa _{e}+\kappa _i$^52^. Furthermore, the output coupling ratio is defined by the coupling parameter $(r_c=\kappa _e/\kappa )$ and the damping rate of the Bogoliubov mode of the BEC is denoted by $\gamma _B$. Here ${\hat{P}}_{in}$ is the BEC atomic field input noise, whose mean value is zero.

Assuming no correlation between any two system operators, i.e., $\langle \hat{a}\hat{b}\rangle = \langle \hat{a} \rangle \langle \hat{b} \rangle$, which is called the mean-field approximation, and supposing that $\langle {\hat{a}}_{in}\rangle =\langle {\hat{a}}_{int}\rangle =\langle {\hat{P}}_{in}\rangle =0$, the mean-value equations of motion for $\hat{a}$, $\hat{P}$, and $\hat{Q}$ from Eqs. (4a–4c) can be obtained as

5a $$\begin{aligned}&\frac{d \langle \hat{a}\rangle }{dt}=-(\kappa + i\delta _{c})\langle {\hat{a}}\rangle - \zeta \langle \hat{Q}\rangle \langle \hat{a}\rangle + \varepsilon _c + \varepsilon _s e^{-i\delta t}, \end{aligned}$$

5b $$\begin{aligned}&\frac{d\langle \hat{P}\rangle }{dt}=-\gamma _B \langle \hat{P}\rangle -(\Omega _{c}+\omega _{sw})\langle \hat{Q}\rangle -\zeta \langle \hat{a}^{\dagger }\rangle \langle \hat{a}\rangle , \end{aligned}$$

5c $$\begin{aligned}&\frac{d\langle \hat{Q}\rangle }{dt}=\Omega _c \langle \hat{P}\rangle . \end{aligned}$$

By eliminating $\langle \hat{P}\rangle$ from Eqs. (5b) and (5c) the dynamical equation of motion for the mean-value of ${\hat{Q}}$ operator of the BEC Bogoliubov mode is obtained as the following second order differential equation

6 $$\begin{aligned} \frac{d^2\langle \hat{Q}\rangle }{dt^2} +\gamma _B\frac{d\langle \hat{Q}\rangle }{dt}+ \omega _B^2 \langle \hat{Q}\rangle = -\Omega _{c}\zeta \langle \hat{a}^{\dagger }\rangle \langle \hat{a}\rangle . \end{aligned}$$

As is seen from Eq. (6) the Bogoliubov mode of the BEC behaves as a driven-damped simple harmonic oscillator with an effective resonance frequency of $\omega _B=\sqrt{(4\omega _{R}+\frac{1}{2}\omega _{sw})(4\omega _{R}+\frac{3}{2}\omega _{sw})}$, which is called the Bogoliubov frequency.

In the case where the signal field amplitude is much smaller than the coupling field amplitude, i.e., $|\varepsilon _s|\ll |\varepsilon _c|$, the steady state solutions to Eqs. (5a) and (6), to the first order of $|\varepsilon _s|$ in the frame rotating at $\omega _c$ can be written as

7a $$\begin{aligned}&\langle \hat{a} \rangle = a_0 + a_{+} e^{-i\delta t} + a_{-} e^{i\delta t}, \end{aligned}$$

7b $$\begin{aligned}&\langle \hat{Q} \rangle = Q_0 + Q_{+} e^{-i\delta t} + Q_{-} e^{i\delta t}. \end{aligned}$$

The right hand side of Eqs. (7a) and (7b) contain three terms corresponding to the first three components of the Fourier expansion which are the steady state solutions at zero, first, and second order of $\varepsilon _s$, respectively oscillating at the coupling laser frequency $\omega _c$, signal field frequency $\omega _s$, and the four-wave mixing frequency $2\omega _c-\omega _s$ in the laboratory frame^36^. Now, by inserting Eqs. (7a) and (7b) in Eqs. (5a) and (6), the zeroth order components can be derived as

8a $$\begin{aligned}&a_0 = \frac{\varepsilon _c}{\kappa + i\Delta }, \end{aligned}$$

8b $$\begin{aligned}&Q_0 = \frac{-\Omega _c\zeta |a_0|^2}{\omega _B^2}, \end{aligned}$$

wherein $\Delta = \delta _{c} + \zeta Q_0$ is the effective cavity detuning. Furthermore, by equalizing the coefficients with the same frequencies on both sides of the equations of motion, the following algebraic equations can be obtained

9a $$\begin{aligned}&\big (i(\Delta -\delta )+\kappa \big ) a_{+} = -i\zeta a_0 Q_{+} + \varepsilon _s, \end{aligned}$$

9b $$\begin{aligned}&\big (i(\Delta +\delta )+\kappa \big ) a_{-} = -i\zeta a_0 Q_{-}, \end{aligned}$$

9c $$\begin{aligned}&\big (\omega _B^2-\delta ^2+i\gamma _B\delta \big )Q_{+} = -\Omega _c \zeta \big (a_0^{*} a_{+} + a_0 a_{-}^*\big ), \end{aligned}$$

9d $$\begin{aligned}&\big (\omega _B^2-\delta ^2-i\gamma _B\delta \big )Q_{-} = -\Omega _c \zeta \big (a_0 a_{+}^{*} + a_0^{*} a_{-}\big ). \end{aligned}$$

Assuming $a_0$ is real, the solutions of Eqs. (9a–9d) are obtained as follows

10a $$\begin{aligned}&a_+ = \frac{1+if}{i(\Delta -\delta )+\kappa -2f\Delta }\varepsilon _s, \end{aligned}$$

10b $$\begin{aligned}&a_{-} = -\frac{a_+^*}{1+i/f^*}, \end{aligned}$$

10c $$\begin{aligned}&Q_{-} = Q_{+}^{*}, \end{aligned}$$

wherein $\chi =\frac{\Omega _c}{\omega _B^2-\delta ^2-i\gamma _B \delta }$ is called the mechanical susceptibility of the BEC Bogoliubov mode and *f* is defined as $f= \frac{\chi \zeta ^2 a_0^2}{-i(\Delta +\delta )+\kappa }$.

Since we are going to derive the amplitude of the cavity output field emanating through the left mirror, we make use of the input-output relation, $\varepsilon _{out} + \varepsilon _{in} = 2\kappa _e \langle \hat{a}\rangle$, where $\varepsilon _{in}$ and $\varepsilon _{out}$ are, respectively, the cavity input and output field amplitudes. Similar to relations (7a) and (7b), the cavity output field amplitude in the rotating frame can be written as follows

11 $$\begin{aligned} \varepsilon _{out} = \varepsilon _{out0} + \varepsilon _{out+} e^{-i\delta t} + \varepsilon _{out-} e^{i\delta t}, \end{aligned}$$

where $\varepsilon _{out0}$ is the central band amplitude of the cavity output field oscillating at the frequency $\omega _c$ while $\varepsilon _{out+}$ and $\varepsilon _{out-}$ are the two sidebands oscillating, respectively, at frequencies $\omega _s$ and $2\omega _c-\omega _s$ in the laboratory frame. Based on Eq. (4a), the input field amplitude is $\varepsilon _{in}=\varepsilon _c+\varepsilon _s e^{-i\delta t}$. If we substitute the input field amplitude as well as $\langle \hat{a}\rangle$ and $\varepsilon _{out}$ from Eqs. (7a) and (11) in the input-output relation, the central band amplitude of the output field is obtained as $\varepsilon _{out0} = 2\kappa _e a_{0}-\varepsilon _c$, while the two sideband amplitudes are determined by $\varepsilon _{out+} = 2\kappa _e a_{+}-\varepsilon _s$, and $\varepsilon _{out-}=2\kappa _e a_{-}$.

The cavity reflected field amplitude oscillating at the signal frequency is defined as the ratio of the cavity reflected field amplitude oscillating at the signal frequency ($\varepsilon _{out+}$) to the input signal field amplitude $(\varepsilon _{s})$, i.e., $\varepsilon _{R} = \varepsilon _{out+}/\varepsilon _s$. In the critical regime, where the rate of the cavity inaccessible loss $(\kappa _i)$ is equal to the loss rate of the intracavity optical field $(\kappa _e)$, the coupling parameter is $r_c =1/2$, and the cavity reflected field amplitude at the signal frequency is obtained as

12 $$\begin{aligned} \varepsilon _{R} = \frac{\kappa (1+if)}{i(\Delta -\delta )+\kappa - 2f \Delta }-1. \end{aligned}$$

Finally, the cavity power reflection coefficient at the signal frequency is defined as the square of the reflected field amplitude at the signal frequency as

13 $$\begin{aligned} R=|\varepsilon _R|^2. \end{aligned}$$

## Results and discussion

In order to investigate how the present hybrid OMS behaves as an optical transistor that can amplify the input signal, we study the behavior of the real part of reflected field amplitude given by Eq. (12), which represents the cavity absorptive behavior at the signal frequency, as well as the cavity power reflection coefficient at the signal frequency (Eq. (13)) in terms of the coupling-signal detuning $\delta =\omega _s-\omega _c$ in the regime where the system is stable.

Here, our results have been obtained based on the experimentally feasible parameters given in Refs.^53,54^. We have considered a cavity with the length of $L=178$ $\upmu$m, damping rate of $\kappa =10^6$ Hz, and bare frequency of $\omega _{0}=2.41494\times 10^{15}$ Hz which corresponds to a wavelength of $\lambda =780$ nm. The cavity contains a trapped BEC formed by $N=10^6$ Rb atoms having a transition frequency of $\omega _{a}=2.41419\times 10^{15}$ Hz. The strength of the atom-field coupling is $g_{0}=2\pi \times 14.1$ MHz, the atom recoil frequency is $\omega _{R}=23.7$ kHz, and the BEC damping rate of the Bogoliubov mode of the BEC is $\gamma_{B} =10^{-4}\kappa$.

In order that the system is on-resonance, we obtain our results in the red detuning regime of $\Delta =\omega _B$ which leads to the following cubic equation in terms of $\omega _{c}$

14 $$\begin{aligned} \omega _0-\omega _c+\frac{1}{2}\frac{N g_{0}^{2}}{\omega _c-\omega _a}-\frac{N g_{0}^{4}\Omega _c}{4\omega _{B}^{2}}\frac{|a_0|^2}{(\omega _c-\omega _a)^2}=\omega _B. \end{aligned}$$

For any specified value of $\omega _{sw}$ the value of $\omega _B$ and subsequently the square of the optical zero-order component, i.e, $|a_0|^2=|\varepsilon _c|^2/(\kappa ^2+\omega _{B}^2)$ are determined based on Eq. (8a). Since Eq. (14) is a third order algebraic equation, it has three roots for each value of $\omega _{sw}$. However, only for one of them the system is stable according to the Routh-Hurwitz criteria^55^.

**Figure 2:**
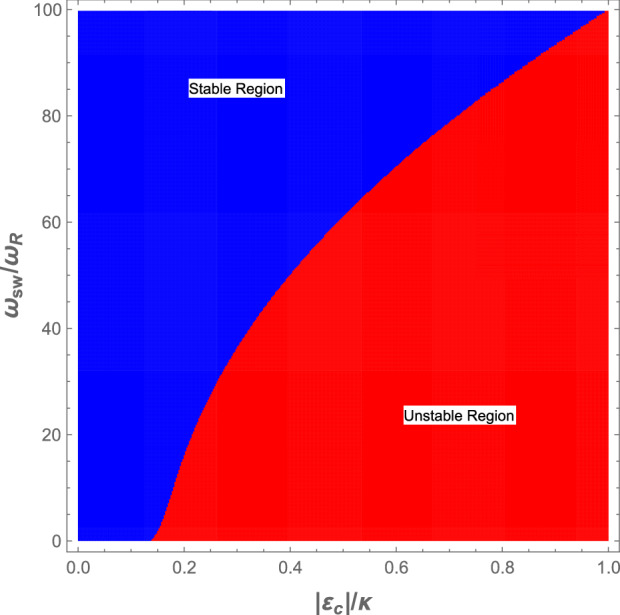
(Color online) The stable (blue) and unstable (red) regions, derived from the Routh-Hurwitz criteria where the horizontal axis is the normalized coupling laser pumping rate ($|\varepsilon _c|/\kappa$) and the vertical axis is the normalized s-wave scattering frequency (${\omega _{sw}}/{\omega _{R}}$). The parameters are given in the second paragraph of section “Results and discussion”.

In Fig. 2, the stable and unstable regions of the system have been indicated based on the Routh-Hurwitz criteria as a contour plot in terms of two experimentally controllable parameters of the system: the normalized coupling laser pumping rate ($|\varepsilon _c|/\kappa$) and the normalized s-wave scattering frequency (${\omega _{sw}}/{\omega _{R}}$). The stable and unstable regions have been shown, respectively, by blue and red colors. Experimentally, the coupling laser pumping rate can be controlled by the power of the coupling laser while the *s*-wave scattering frequency of atomic collisions can be manipulated by the transverse frequency of the optical trap of the BEC?. Figure 2 shows that for $|\varepsilon _c|\ge 0.14 \kappa$ the *s*-wave scattering frequency is bound to have a nonzero minimum value so that the system remains in the stable regime, where the mentioned minimum of $\omega _{sw}$ increases by increasing $|\varepsilon _c|$. On the other hand, for any fixed value of the *s*-wave scattering frequency, $|\varepsilon _c|$ should be lower than a maximum value so that the system is stable.

**Figure 3:**
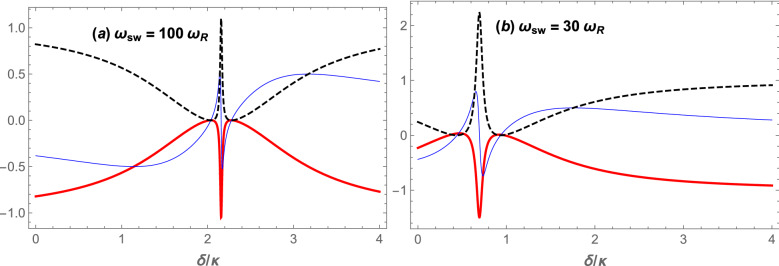
(Color online) $\mathrm Re[\varepsilon _R]$ (red thick curve) and $\mathrm Im[\varepsilon _R]$ (blue thin curve) as well as the cavity power reflection coefficient *R* (black dashed curve) at the signal frequency versus the normalized frequency detuning ($\delta /\kappa$) for the coupling laser pumping rate of  $|\varepsilon _c| = 0.10\kappa$, in the red detuning regime of $\Delta =\omega _B$ for two different values of the *s*-wave scattering frequency: (**a**) $\omega _{sw}=100\omega _{R}$, and (**b**) $\omega _{sw}=30\omega _R$. The other parameters are the same as those of Fig. 2.

To study the mechanism of the system response to the input signal, we have plotted in Fig. 3 the real and imaginary parts of the output (reflected) field amplitude as well as the cavity power reflection coefficient at the input signal frequency versus the normalized frequency detuning ($\delta /\kappa$) when the system is in the red detuning regime of $\Delta =\omega _B$. Here the coupling laser pumping rate has been fixed at $|\varepsilon _c| = 0.10\kappa$ for two different values of the *s*-wave scattering frequency of atomic collisions: $\omega _{sw}=100\omega _{R}$ (Fig. 3a), and $\omega _{sw}=30\omega _{R}$ (Fig. 3b). For each value of $\omega _{sw}$ the effective frequency of the Bogoliubov mode of the BEC, which plays the role of the mechanical mode frequency in the present hybrid OMS, is determined to be $\omega _{B}\approx 2.16\kappa$ in Fig. 3a and $\omega _{B}\approx 0.72\kappa$ in Fig. 3b. For each mentioned value of $\omega _B$ one can solve Eq. (14) to find three roots for $\omega _c$ which just one of them is acceptable based on the stability condition of the Routh-Hurwitz criteria. In this way, in an experimental setup with specified values of $\varepsilon _c$ and $\omega _{sw}$, the right value of the coupling laser frequency $(\omega _c)$ can be determined so that if the cavity is driven at that frequency, the system response to the input signal appears as those demonstrated in Fig. 3a and 3b. Here, the three roots of Eq. (14) are approximately given by $2.41494\times 10^{15}$, $2.41419\times 10^{15}$, and $2.41418\times 10^{15}$, where the first root satisfies the stability conditions. In this way, the right value of the coupling laser frequency is $\omega _c\approx 2.41494\times 10^{15}\mathrm Hz$.

In the present system, the input signal acts as a time-dependent perturbation (the last term in the Hamiltonian of Eq. (3)) where the absorptive and dispersive responses of the system to it are manifested, respectively, in the real and imaginary parts of the output (reflected) field amplitude. Since in Fig. 3a and b the enhanced effective optomechanical coupling $\zeta a_0<\kappa /2$ the system is in the OMIT regime^56^ where a fairly narrow transparency window appears at $\delta =\omega _B$. That is why the dip of the transparency window of the OMIT in Fig. 3a and 3b occurs, respectively at $\delta \approx 2.16\kappa$ and $\delta \approx 0.72\kappa$. Besides, since at the center of the transparency window $\mathrm Im[\varepsilon _{R}]=0$ and $\mathrm Re[\varepsilon _{R}]\le -1$, the power reflection coefficient is determined by $R=|\mathrm Re[\varepsilon _{R}]|^2$, which is in the range of $R\ge 1$. When the maximum value of the power reflection coefficient at the signal frequency is greater than one, the phenomenon of OMIG^45^ happens and the cavity acts as an amplifier^57^.

Since $\omega _{B}$ is an increasing function of $\omega _{sw}$, for large values of $\omega _{sw}$, like the case of Fig. 3a, the system is in the resolved sideband (RSB) regime, where $\omega _{B}>\kappa$, while for small values of $\omega _{sw}$, like the case of Fig. 3b, the system is in the URSB regime, where $\omega _{B}<\kappa$. In this way, the position of the dip of absorptive response (the red curves) indicates whether the system is in RSB or URSB regimes. Therefore, the system regime can be switched from RSB to URSB or vice versa by manipulating $\omega _{sw}$ which itself is controllable through the transverse trapping frequency of the BEC^48^. As is seen from Fig. 3a, where the system is in the RSB regime, $\mathrm Re[\varepsilon _{R}]\approx -1$ and consequently $R\approx 1$ which corresponds to the situation of the standard OMIT where the reflection of the input signal is nearly $100\%$. However, in the URSB regime $\mathrm Re[\varepsilon _{R}]<-1$ and consequently, $R> 1$ which corresponds to the OMIG in which the input signal is amplified in the output of the system. As is seen from Fig. 3b the input signal power can be increased more than two times in the output of the cavity since $\mathrm Re[\varepsilon _{R}]<-2$ and consequently $R> 2$.

**Figure 4:**
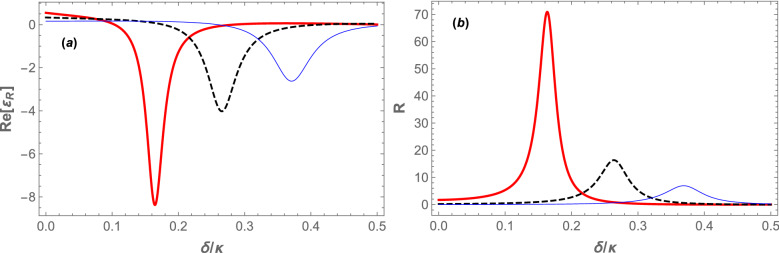
(Color online) (**a**) The real part of the reflected field amplitude. (**b**) The cavity power reflection coefficient at the signal frequency, versus the normalized frequency detuning ($\delta /\kappa$), for the coupling laser pumping rate at $|\varepsilon _c| = 0.10\kappa$ and three different values of $\omega _{sw}$, in the red detuning regime where $\Delta =\omega _B$. The red thick, black dashed, and blue thin plots correspond to the values of $\omega _{sw}=5\omega _{R}$, $\omega _{sw}=10\omega _{R}$ and $\omega _{sw}=15\omega _{R}$, respectively. The other parameters are the same as those of Fig.  2.

In Fig. 4, we have plotted the real part of the reflected field amplitude of the system (Fig. 4a) and the cavity power reflection coefficient (Fig. 4b) at the signal frequency versus the normalized coupling-signal detuning ($\delta /\kappa$) for $|\varepsilon _c|=0.10\kappa$ and three different values of the *s*-wave scattering frequency: $\omega _{sw}=5\omega _{R}, 10\omega _{R}$, and $15\omega _{R}$ represented, respectively, by red thick, black dashed and blue thin curves. It should be emphasized that for $|\varepsilon _c|=0.10\kappa$ the values of $\omega _{sw}$ have been so considered that the system is in the stable (blue) region of Fig. 2. Each curve in Fig. 4a, which is a dip of the OMIT transparency window like that of the absorptive response (red thick curve) in Fig. 3, corresponds to a specified value of $\omega _{sw}$ for which the cavity is driven at the specified value of $\omega _c$ determined based on Eq. (14). Figure 4b shows that there exists a peak of power reflection coefficient corresponding to each dip in Fig. 4a. Since all the dips of the OMIT transparency windows in Fig. 4a occur at $\delta =\omega _B<\kappa$, the system is in the URSB regime in which it acts as an amplifier. As is seen, the deeper is the dip of the transparency window, the larger is the peak of the power reflection coefficient of the system (the more amplification) so that for $\omega _{sw}=5\omega _R$ the amplitude of the input signal is amplified up to 70 times in the output of the cavity, i.e., $7000\%$ amplification.

**Figure 5:**
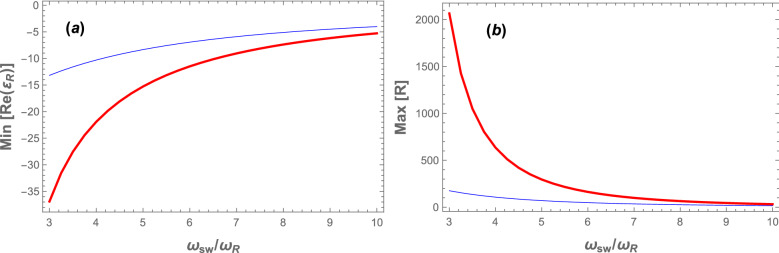
(Color online) (**a**) The minimum value of the real part of the reflected field amplitude, (**b**) the maximum value of the cavity power reflection coefficient at the signal frequency versus $\omega _{sw}/\omega _{R}$ for two different values of the coupling laser pumping rate: $|\varepsilon _c|=0.10\kappa$ (the blue thin curve) and $|\varepsilon _c|=0.15\kappa$ (the red thick curve). The other parameters are the same as those of Fig. 2.

To see how the amplification capacity of the system can be enhanced, in Fig. 5a we have plotted the minimum of the real part of the reflected field amplitude (corresponding to the minimum values of OMIT dips of Fig. 4a which occur at $\delta =\omega _B$ for every $\omega _{sw}$), and also we have plotted in Fig. 5b the maximum values of the power reflection coefficient at the signal frequency (corresponding to the maximum values of peaks of Fig. 4b) versus $\omega _{sw}/\omega _{R}$ for two different values of coupling laser pumping rate: $|\varepsilon _c|=0.10\kappa$ (the blue thin curves), and $|\varepsilon _c|=0.15\kappa$ (the red thick curves). It should be noted that the variation range of $\omega _{sw}$ in Fig. 5 has been chosen so that the system is stable and in the URSB regime. According to Fig. 5a, by decreasing $\omega _{sw}$ the negativity of the real part of the reflected field amplitude increases, and consequently the maximum power reflection coefficient (amplification capacity) increases for each value of $|\varepsilon _c|$. Furthermore, as is seen from Fig. 5b at any $\omega _{sw}$ the maximum value of the power reflection coefficient has a larger value for a larger $|\varepsilon _c|$ (compare the red thick and blue thin curves). For example, at $\omega _{sw}=3\omega _{R}$ the maximum value of the power reflection coefficient is more than 2000 (corresponding to $2\times 10^5\%$ amplification) for $|\varepsilon _c|=0.15\kappa$, while it is about 200 (corresponding to $2\times 10^4\%$ amplification) for $|\varepsilon _c|=0.10\kappa$.

**Figure 6:**
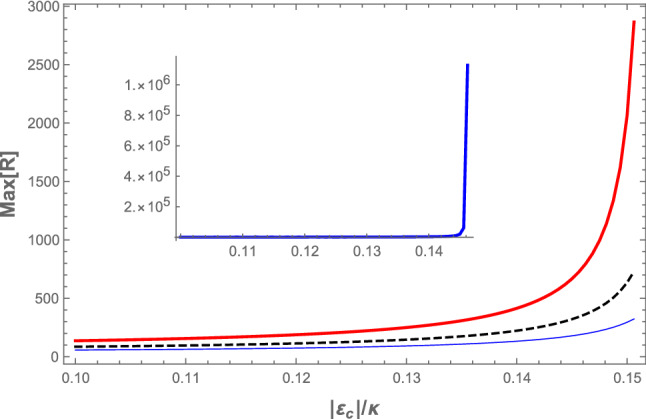
(Color online) The maximum value of the cavity power reflection coefficient at the signal frequency versus the normalized coupling laser pumping rate ($|\varepsilon _c|/\kappa$) for different values of the *s*-wave scattering frequency: $\omega _{sw}=3\omega _{R}$ (red thick curve), $\omega _{sw}=4\omega _{R}$ (black dashed curve) and $\omega _{sw}=5\omega _{R}$ (blue thin curve). The inset shows $\mathrm Max[R]$ versus $|\varepsilon _c|/\kappa$ for $\omega _{sw}=\omega _{R}$. The other parameters are the same as those of Fig. 2.

Furthermore, to show explicitly how the maximum value of the power reflection coefficient at the signal frequency increases by increasing the coupling laser pumping rate, in Fig. 6, we have plotted $\mathrm Max[R]$ versus the normalized coupling laser pumping rate ($|\varepsilon _c|/\kappa$), for three different values of the *s*-wave scattering frequency: $\omega _{sw}=3\omega _{R}$ ( red thick curve), $\omega _{sw}=4\omega _{R}$ (black dashed curve), and $\omega _{sw}=5\omega _{R}$ (the blue thin curve). The inset of Fig. 6 shows $\mathrm Max[R]$ versus $|\varepsilon _c|/\kappa$ for $\omega _{sw}=\omega _{R}$. As is seen from Fig. 6, $\mathrm Max[R]$ increases very rapidly by increasing the coupling laser pumping rate where the system gets near to the boundary of the instability region for each $\omega _{sw}$ (the red region in Fig. 2). Besides, this increase is more intense for lower values of $\omega _{sw}$ so that for $\omega _{sw}=\omega _{R}$ (the inset of Fig. 6) the maximum value of the power reflection coefficient at the signal frequency exceeds $10^6$ before the system enters the instability regime. It means that in the red detuned and URSB regime, the present hybrid OMS can amplify the input signal more than $10^8\%$ while it is in the stable regime. This result is much greater than those obtained in the previously proposed schemes^36–41,45^, and specifically three orders of magnitude larger than that obtained in Ref.^39^, where it has been shown that in the hybrid OMS (in which the effect of atom-atom interaction is neglected and no stability check has been presented) the maximum amplification that can be achieved is less than $2\times 10^5\%$ in the blue regime of optomechanics, where the system has usually stability problems.

Finally, it is worth reminding that another scheme for the generation of gain in OMSs has been already proposed based on the coherent modulation of the mechanical oscillator^58^. Such a scheme can lead to the generation of Casimir photons and phonons^59^ which makes the system act as a quantum amplifier or squeezer^60^ or behave as a quantum sensor for weak force sensing below the standard quantum limit (SQL)^61–63^. As an interesting outlook for future researches, one can investigate the possibility of manifestation of OMIG in bare or hybrid OMSs whose mechanical modes are coherently modulated in the URSB regime. One of the best methods for studying the response of such driven-dissipative systems is the approach of Green’s function and the linear response theory^64–68^which have been recently attracted much attention.

## Summary and conclusions

We have studied the phenomenon of OMIG in a hybrid OMS consisting of a one dimensional BEC whose cavity is driven by a coupling laser tuned to the red sideband of the cavity in order to generate an effective optomechanical coupling between the optical mode of the cavity and the Bogoliubov mode of the BEC. It has been shown that if the cavity is exposed to a weak input signal behaving as an external signal laser which drives the intracavity mode perturbatively, the system acts as an optical transistor that can magnify the input signal in its output while the system is in the URSB regime. For this purpose, we have solved the Heisenberg-Langevin equations to obtain the reflected field amplitude in the cavity output whose real and imaginary parts represent, respectively, the absorptive and dispersive response of the system to the input signal. We have also shown that the system can switch from RSB to URSB by manipulating the *s*-wave scattering frequency which itself can be controlled by the transverse trapping frequency of the BEC. More importantly, we have shown that the amount of the system amplification can be enhanced and controlled by the coupling laser power as well as the *s*-wave scattering frequency as far as the system is stable and in the URSB regime. It has been shown that by decreasing the *s*-wave scattering frequency from one hand, and by increasing the coupling laser power from the other hand, the cavity power reflection coefficient at the signal frequency can be enhanced considerably before the system enters the instability region. Based on our obtained results, the input signal can be amplified more than $10^8\%$ which is much larger than those obtained in previous studies.

## Data Availability

All data that support the findings of this study are included within the article.
